# “Embracing is the most important thing we can do” – Caring for the family members of patients at risk of suicide

**DOI:** 10.1080/17482631.2021.1996682

**Published:** 2021-11-21

**Authors:** May Vatne, Vibeke Lohne, Dagfinn Nåden

**Affiliations:** Faculty of Health Sciences, Oslo Metropolitan University, Oslo, Norway

**Keywords:** Suicidal inpatients, Family members, Mental health services, Health personnel’s experiences, Acknowledgement, Embracing, Involvement, Collaboration

## Abstract

**Aim:**

This study explores mental health personnel’s experiences in the encounter with family members of patients at risk of suicide so as to develop a deeper understanding of the content of caring.

**Methodology:**

Data were collected using semi-structured interviews with 12 participants and were analysed and interpreted using a model inspired by the philosophical hermeneutics of Gadamer. The context was psychiatric wards.

**Findings:**

Through a thematic analysis, four themes emerged: Acknowledgement as a premise for involving family members. Embracing with the family members’ feelings and reactions. Strengthening hope in a situation entailing a serious risk of suicide. Providing reassurance to family members in transitional situations.

**Conclusion:**

Witnessing the family members’ suffering and needs is understood as arousing a sense of responsibility in the participants and triggering various care strategies such as listening, embracing, strengthening hope and providing reassurance.

## Introduction

Every year, some 800,000 people die by suicide, and families, friends, schoolmates, colleagues and communities are left bereaved and often without assistance (WHO, World Health Organization, 2018). In Norway, where this study was done, 671 people died by suicide in 2018, similar to figures in Europe, North America and Australia (NIPH, Norwegian Institute of Public Health, 2020). Figures from the corona year 2020 showed 639 deaths in Norway by suicide and self-harm (NIPH, Norwegian Institute of Public Health, 2021).

A high proportion of patients admitted to the acute psychiatric ward have contemplated or attempted suicide, and assessment of suicide risk is a key task in such wards (Mellesdal et al., 2010). Contact with health personnel may give suicidal patients a feeling of being acknowledged and valued (Berg et al., 2017; Hagen et al., 2018; Vatne & Nåden, 2016). Health personnel who work as milieu therapists provide 24-hour care for suicidal patients and are primary contacts for patients in collaboration with psychiatrists and psychologists. Milieu therapists are also the personnel relatives normally meet when they call or visit the patient on the ward. The topic of this study is health professionals’ experiences from encounters with family members of patients at risk of suicide, in the context of Norwegian psychiatric wards.

The risk of suicide challenges the ideal of self-determination and health legislation perhaps more than anything else; particularly if the suicidal patient does not want help to continue living and will not consent to health care, whereas the duty of healthcare professionals, according Norwegian legislation, is to save lives (Larsen & Pedersen, 2017). Although the primary focus of health professionals is on the patient, their professional, ethical and legal responsibilities include those the patient considers his/her family members. Norwegian health legislation obliges health personnel to provide information and enable co-determination. The legislation gives patients the right to decide for themselves whether individual relatives are to be considered a family member, a friend, neighbour, distant relative or other, and what information can be provided to them about him/her (Bøckmann & Kjellevold, 2015).

Living close to a person at suicidal risk may be painful and demanding. Persons in suicidal suffering struggle with feelings such as despair, loneliness, anxiety, shame, hopelessness, and the wish to continue life but also a longing to escape suffering (Beskowl et al., 2013; Brown et al., 2005; Shneidman, 1998; Williams, 2014). Research reveals a high degree of comprehensive care burdens and suffering in families living with a person at risk of suicide (Chiang et al., 2015; Fan-Ko & Long, 2008; Lachal et al.’s, 2015). McLaughlin et al. (2014) found that family members were in a state of exhausting responsibility and stress. They struggled with feelings of helplessness and guilt over not having been able to effectively help the suicide victim, and they were affected by the reality that the person they had wanted to help kept secret and was ashamed of his or her own suicide ideation. According to Sun et al. (2013), psychoeducation enhanced the family members’ caregiving capacity, because they developed a better understanding of the suicidal person. The tools they acquired, however, did not reduce the stress they experienced.

Family members have different roles for the patient, such as caregiver, source of knowledge, representative and a key member of the patient’s local support environment. The family members also need support to fill these roles. Proper involvement is important for both patients and the families, including situations when the influence is of a negative nature (Bøckmann & Kjellevold, 2015; Pedersen & Weimand, 2017). Family members of patients at risk of suicide want to be included in the patient’s treatment process (McLaughlin et al., 2016; Sellin et al., 2017). Related to patients at risk of suicide, Le Moal et al. (2018) point out that a key component in the patient’s network—“the caregiver”—has not been included in prevention strategies. In a recent action plan for suicide prevention in Norway, however, one of the proposed measures is to strengthen the focus on family members as a resource in suicide prevention, and on their need for support and information (Norwegian Ministry of Health and Care Services, 2020).

The family members` capacity to assess and respond to the problems of a person close to them may be of crucial importance in the prevention of suicide (Owens et al., 2009), but they experience that the needs they deem necessary to enable them to perform their duties as caregivers, such as advice, support, acknowledgement and information, are not satisfactorily met (McLaughlin et al., 2016). Research is scarce on health professionals’ experiences of how to meet family members’ needs and strengthen their capabilities.

The aim of this study is to investigate health personnel’s experiences in the encounter with family members of patients at risk of suicide to develop a deeper understanding of the content in providing care for family members. The research question is: How do health professionals describe and understand their responsibilities and tasks in their encounter with family members of patients at risk of suicide?

## Methodology

The study is inspired by Gadamer’s philosophical hermeneutics (2004). Hermeneutical understanding has been a guiding principle in the encounter with both the participants and the texts. Hermeneutics is characterized by sensitivity and receptiveness to what reveals itself in the process of discovery, which in turn can engender new questions so that existing understanding is challenged and expanded (Gadamer, 2004; Nåden, 2010). In a dialogue in which both the researcher and the interviewee are engaged, the case is principally inexhaustible: “we can always only understand better” (Fog, 2004, p. 208). The researchers’ own pre-knowledge could represent an obstacle, while at the same time, pre-knowledge represents a positive premise for new understanding (Gadamer, 2004). Although we argue that the first author’s pre-understanding through working with suicidal patients in a context of mental health prevention has a positive impact on the conduct of the study, it is crucial to be critically aware of one’s pre-understanding throughout the research process.

In a theoretical perspective of caring ethics, suffering is part of being human. Suffering affects the human being’s existence and meaning, and this includes both the patient and the family relative. The experience of not being seen by others, of being considered “dead”, produces an intense feeling of loneliness and, according to Eriksson, is perhaps the deepest form of suffering a person can experience (Eriksson, K, 2006, 2018, p. 328). Seeing the other person is one of the components of acknowledgement. In this study, the concept of acknowledgement is understood as valuing the other for what he/she is and experiencing his/her innermost being (Schibbye, 2009). As Schibbye points out, acknowledgement is more about recognizing, discriminating, consolidating and strengthening than about confirmation, empathy and compassion. Suffering causes hopelessness, but also engenders hope based on a belief and trust in life itself, which is vital for alleviating suffering (Eriksson, K, 2018). Hope is a key concept in the encounter with suicidal patients (Cutcliffe & Barker, 2002; Herrestad, 2009; Moore, 2005). Caring ethics is expressed primarily as a moral practice aimed at taking care of the other so that courage and hope are strengthened (Martinsen, 2005).

### Participants, context, and recruitment

Twelve health personnel—three men and nine women—were included in the study. The participants comprised one social educator, three social workers and eight nurses, all having specialized studies in mental health work. Several of the participants had further education in other fields. The average sum of work experience in mental health was 15 years, primarily in the specialist health service and acute psychiatric wards; most participants had lengthy experience in the institution where they were employed. All participants were experienced in having contact with family members: parents, mainly mothers, spouses, siblings, children, and some other relatives. Factors such as gender, education, experience in mental health work and contact with family members of patients at risk of suicide were the inclusion criteria for participation in the study. Apart from three participants in different special functions, all worked as milieu therapists. The participants belonged to a total of six different wards at the two hospitals.

The request to interview health personnel was sent to the senior clinical leader in two hospitals, both of which had acute psychiatric functions. The leader made sure that everyone who met the criteria for participation received information about the project and could register their interest. One of the researchers received the names of interested parties either from employees themselves or from one of the staff members who had a coordinating role.

### Data collection

The data material consists of texts from individual qualitative research interviews, audio-file recorded and transcribed by a company certified to do this. The interviews lasted between 60 to 90 minutes each. With one exception, the interviews were conducted at the participants’ workplace. The interviews were prepared and carried out in accordance with Kvale’s conception of the interview as a research conversation (Kvale & Brinkmann, 2009); they were conducted based on a guide having six main questions. Some adjustments were made in consultation with three clinical leaders at different levels at one of the institutions prior to data collection during a meeting at which the questions were introduced.

The opening question invited the participants to relate a situation in which they had an encounter with family members of a patient who was followed up because he/she was at risk of suicide or had attempted suicide or had succeeded in taking his/her own life. The further questions concerned their thoughts about relatives as a resource, what they think about care for family members of suicidal patients, what they perceive as especially challenging and as dilemmas in collaboration with families, how they try to give patients and family members a sense of security during discharges and transitional periods. The guide also contained one question that opened for providing information that the other questions did not capture.

### Thematic analysis

All texts were reviewed based on the questions in the interview guide, and the content that was understood as responding to these was presented in a resource group affiliated with the project. The researchers individually read the interviews to familiarize themselves with the texts and with what the participants intended to convey; this is what Braun & Clarke (2006) call the first phase of a thematic analysis. In the quest for themes that might illuminate the research question, the individual interviews were then reviewed by the researchers together as a group in order to share ideas about potential themes. At the same time, text passages were marked when they were deemed appropriate to document various themes. Already in this phase, “Acknowledgement” came up as a potential theme, along with the terms “Embracing”, “Hope”, and “Safe discharge”; these remained constant as key categories in what would become the findings of the study. In this process, it became clear that major portions of the texts concerned contact with family members in cases where the patient succeeded in taking his/her own life. These passages in the texts were extracted for a separate article. During the work to present results, the themes became more distinct and were assigned a name. “Defining and naming themes», according to Braun & Clarke (2006), Braun & Clarke 2006 constitutes a separate phase in the analytical process. Table I illustrates the process of searching for potential themes and leading up to findings, exemplified by the theme “Embracing with the family members’ feelings and reactions”. A hermeneutical approach to the text entails remaining receptive to a new understanding, until the presentation is understood as consistent and the statements that are selected seem to give meaning to and substantiate the themes. The phase of presenting the results is described by Braun & Clarke (2006) as the last phase in the thematic analysis.

**Table I.** An example from phases in the analysis process

Searching for themes	Checking themes in relation to the coded extract	Naming themes
- Listening to what is offered - Accepting despair, worries, hopelessness, exhaustion, frustration over systems - Withstanding and persevering through the ordeal of suffering - Being available—physically and mentally	… meaning to allow the family members initially to purge themselves entirely and to respond to feeling with feeling, and to simply accept everything the person has to say and merely respond by taking input, but without offering any professional input of one’s own. Information will come at a later stage; first, think shock and crisis, rather than inundating the person with information. Embracing with and reassure the person.	Embracing with the family members’ feelings and reactions

The project was led by the first author, who also conducted the interviews. The co-authors participated in the project from planning to publication of the results. The project has had an external resource group consisting of participants representing patient and user’s experiences, family member’s experiences, clinical experiences and research. The group had consented to follow the project and have offered input, via regular meetings with the researchers.

### Research ethics

Although the participants were cautious about not identifying patients, family and staff members by name and personal details, total observance of the duty of confidentiality will always be a challenge in research involving questions about personal experiences. Information that might help identify persons was therefore either omitted or anonymized, even at the risk of losing some of the meaningful content. In a research interview, some moral responsibility is also a given necessity (Fog, 2004), since openness about personal experiences requires the researcher to be sensitive to the participant’s integrity.

The study was approved by the Ombudsman for Privacy of the Norwegian Social Science Data Services, no. 54,982/2017. The participants gave written consent, based on written and oral information they received about the project, including anonymization and the right to withdraw from the study.

## Findings

Health personnel’s experiences with and perceptions of opportunities and challenges in the encounter with family members of patients at risk of suicide were categorized in four themes: 1) Acknowledgement as a premise for involving family members, 2) Embracing with the family members’ feelings and reactions, 3) Strengthening hope in a situation entailing a serious risk of suicide, and 4) Providing reassurance to family members in transitional situations. The findings are presented and documented by summarized texts and selected quoted passages that illustrate the themes.

### Acknowledgement as a premise for involving family members

Acknowledgement is understood as a basic attitude held by the participants and as a premise for the way they relate to family members of patients hospitalized because of suicidal ideation or attempted suicide. Acknowledgement appears in descriptions of encounters characterized by openness, listening, understanding, respect, confirmation, and equality. Providing support to alleviate the family members’ frustration is described as an important action. One of the participants said:

If one manages to acknowledge the ordeal that family members experience in a trustworthy way, then I think that this functions as comfort.

The descriptions are understood as expressions of a respectful and humble attitude towards the decisions and actions of the relatives as a consequence of the ordeal they are going through, often over time. The perseverance of parents—particularly the mothers—when patients are repeatedly admitted to hospital, is emphasized as recurrent: “They return and are admitted as twenty-year-olds, and then as thirty-year-olds, and it’s always the parents who are still there for them”. The participants describe having respect for the choices relatives make, including those that do not necessarily lead to any change in the patient, such as the following reflection illustrates:

How can I tell what is best? I can ask questions, like: Are there other ways of looking at this, but I don’t have a right to say ‘Well, you can’t do this or that’. Would I have been able to withstand and to refrain from getting over-involved if this was my own son?

Being listened to and having someone who is open to family members’ experiences based on their interaction with the patient is described by participants as providing new insights to inspire new approaches to treatment and planning of follow-up after admission to the psychiatric ward. “Our knowledge about the patient is largely ‘pathologically dependent’. The family members have varying degrees of knowledge about the patient, but it far surpasses what we know”, one participant said. One of them relates a conversation with a family member whose spouse had been admitted after attempted suicide, and expressed concerns related to alcohol abuse. This concern led to a talk about that, the participant recalled, and that there was also a sort of budding realization on the part of the patient that there was maybe an unfortunate relationship with intoxicants, in this case alcohol. Another participant recalled a conversation with some younger siblings of a patient at risk of suicide. The siblings asked questions about self-harm, a topic that had not been discussed with the patient. “So, when that came up, I was a little caught off guard, and I answered honestly and said that I didn’t know”, the participant said. In a subsequent conversation when the patient was present, the topic came up, and the patient was also “caught off guard”. The conversation calmed the situation for the siblings, and it was a revelation for the participant, because the health personnel realized that self-harm was a subject that needed to be discussed, in addition to the risk of suicide. This conversation provided insight into the siblings’ worries about the patient, but also into the worries they had about their own safety and about the lives of their schoolmates, the participant said.

Acknowledgement also calls for understanding, confirmation and equal status. The participants are encouraged to consider the point of view of the family members and to provide care both to the patient and to family members. “Family members of patients are like most people”, one participant says; “they are not always a resource”. Several examples are given of cases involving complicated relationships and unfortunate dynamics, particularly between young girls and their mothers, where threats of suicide and repeated hospitalization because of intoxication are the main topics. Not infrequently, the mothers are also mentally unbalanced. But they still call each other, even though the health personnel have advised the patient to not do that because the patient seems to gets more ill from it.

However, as one participant stated:

The way I see it, or know, is that some of the staff sometimes think it helps the patient if you keep a difficult relative away from them. … But I think, even though they aren’t a positive resource by definition, get them in here! Maybe things will improve. Because family members are important regardless.

## Embracing with the family members’ feelings and reactions

“Embracing the family members is the most important thing we can do”, several of the participants emphasize. The term “embracing” is stressed in all the interviews and is understood in terms of content as working towards listening, being receptive to, embracing and tolerating different feelings and reactions, as worries how things will turn out. Then, a participant stated, “It’s often just a matter of embracing, of supporting them. You can’t remove the burden from them, really … They have been carrying it, and it is there.”

Assuming the burden of the emotional pressure may alleviate and relieve the pain, like the fear of recurrence after a loved one survived two serious suicide attempts, as one participant discusses. The patient is cared for, “but no one asks the wife how she manages to live with this”, this participant said. Another tells about a family member who has moved in with a parent to try to control a dramatic situation. Then he calls the emergency ward and says that he can’t take it any longer:

So, I support him and say that it sounds like he has too much to handle and that he needs to contact the casualty clinic. He obviously did this, because the next day, the patient herself was admitted here. The family members were exhausted. He hadn’t slept for the past couple of weeks, and he has a job and children to look after.

Not all family members ask for a conversation. It’s a matter of seizing opportunities and paying attention to them when you accompany them off the ward after a visit, the interviewee says: “Do they look ‘tense’? Do they need to have a talk right now?” The participants talk about family members who are incredibly worn down. “So, in the first meeting, you just have to sort of lend an empathetic ear and support them in the troubled situation they find themselves in at the moment”, one participant says. Family members are persons having differing needs and reactions, and not everyone wants to be deeply involved in the treatment, the participants explain. There are situations in which family members, particularly of patients with a history of repeated admissions, are exhausted and need a break; they don’t need to get so much information. “Sometimes that might be right, but in that case, you need to talk about it so that both parties agree”, one of the participants says. Some of them maybe need to be relieved of responsibility so that they can calm down:

It’s all right to feel that what they are going through is a heavy burden, and we try to reassure them and say that now it is our turn to take care of the patient. We tell them it is all right to stay home one evening and sleep, take care of yourself; do you have someone to talk to? We get in touch with them, or they ring us. We are always here; we are on the supply side.

The participants relate situations when the family members’ reactions reveal negatively charged feelings and reactions associated with the treatment, with persons and systems. They emphasize that there is a lack of resources and of guaranteed service, to ensure that all family members receive help:

If they could be systematically offered several conversations, more regularly, as it says in the guide relating to family members: «predictable contact», then their pressure and burden might have been lessened, and for the patient, too, if we could offer more assistance in helping family members.

The participants describe a procedure of first embracing with and caring for the family relative, and then providing information and guidance. The relatives might start out by saying that they want a lot of information, “but that is often not what they want at all; they really want to tell us how their ordeal has been, to be heard, to be supported”, one participant says. A general explanation of how things usually are when a patient is admitted in this kind of situation looks normally enough, she told, but “I can’t actually say what is enough for them … But we notice, in a way, that they have a need to talk.”

Family members are relieved when a patient has been officially admitted; “they want the patient to be cared for, of course; that is easy to understand.” Disagreements do arise in various situations, for example, when the family member’s opinion is contrary to the polyclinical treatment regimen that is deemed to be a better treatment for young patients with long-term suicidality; the ideation often gets worse when they are kept on the ward for a long period of time. Most often it is these patients, the ones who are repeatedly admitted after ingesting substances or making suicide threats, who refuse contact with their family members. Several participants emphasized this as the most difficult thing: “the fact that we are not allowed to say much to them, and the family are very concerned about the patient.” Some patients do not want the family members to know anything about them; others express a desire to protect their family by denying the staff permission to divulge information, and the staff has to respect this, one of the participants says. They tell the patients that it is a routine procedure to inform family members. When patients impose limitations like that, it makes caregiving for the patient more difficult, the participants think. Just telling the fact so that they don’t have to go around worrying and maybe contacting the police. As one says:

That is really the first step, telling them that the patient is here. Then we can give them a little general information, about visiting hours, what types of treatment we provide and things like that, which can be good care for the family members.

In situations where the patient does not want to reveal the names of family members, the question is repeated a few hours later or the day after; the interviewee motivates the patient by talking about the importance of the patient and relatives getting “in position with one another”, as one of them puts it. It is rarely that we do not get a name when the situation has calmed down, they tell, “and I have never actually seen that anything bad has resulted because of that”, one participant says.

## Strengthening hope in a situation entailing a serious risk of suicide

The participants describe difficulties in striking a balance between conveying the hope and belief that the patient will get better, without under-communicating the seriousness of the risk of suicide. “Letting family members hear that we have much experience handling these kinds of situations can help”, one of them says, and adds:

It is difficult right now, of course. Difficult and saddening, but at the same time you know, at least with some certainty, that things will get better. You try to strengthen hope, and frequently we see that things do indeed get better, too, because they normally do, fortunately … You can be quite certain that things will go well, in a way. … And then, with medication and conversations and reassurance and all the rest, things do get better.

In many situations, the participants’ contact with the patient family members is short-term. “The most difficult thing is to strengthen long-term hope, I think”, one of them says, “This is a limitation of the emergency ward.” Anyway, the participant points out: “We need to work with giving that hope.”

The participants describe how they emphasize specific factors that can raise the patient and family members up out of their hopelessness, as one of them puts it:

We see and focus on and convey the small steps the patient is taking, maybe contrast the previous evening with the situation today. And we do the same thing with the relatives – identify the small steps, the progress we see. It is a matter of remaining realistically ‘tuned in’, but still pull the situation up out of hopelessness, to some extent. However, it is hard not to be overeager. On their behalf, that is. It takes time. We have to sort of take whatever time is required, while at the same time, we have to find resources and forge them, as we say.

The family members’ hope can be positively influenced by information, but information can also exacerbate hopelessness, one participant thinks. “One thing we should do more of on the ward is focus on resources rather than on statistical risks associated with the diagnosis.” Because, as this participant, who has lengthy experience with groups of immediate family members, puts it: “if there is one thing family members need, it is hope.” The participants are keen on providing brief and concise information that allows for a degree of speculation, because the family members often are in crisis. “Through the treatment, the patient will be better”, one participant says.

Perhaps the most demanding aspect, according to several interviewees, is speaking with a mother about her son or daughter’s risk of suicide without disheartening her, as one participant puts it:

Having to say, “There is a high risk of suicide here, and the risk has been there for many years now. You have to care for yourself. Draw some lines.” That is difficult. I think those are the most difficult cases we have been involved in.”

Reflecting on the balance between providing facts that might offer family members hope, but conversely may cause increased worry or hopelessness, a participant says:

It may be unconscious on our part that we don’t inform the family members about suicide risk assessment, out of a desire to protect them. I think that we could be better at talking generally about the risk of suicide, even when it is not present – it should be part of our routine procedure. Sometimes the family members bring up the subject themselves. But at other times, maybe they should not have to relate to that topic; they might be completely exhausted by it and need a break.

## Providing reassurance to family members in transitional situations

The transitional phase, when a patient has been considered at risk of suicide, was a theme in the interviews. The first temporary leave or discharge from the emergency ward is described as a situation often creating uncertainty and worry for the patient, family members and the participants themselves. “Leaves of absence are challenging for the staff as well. One of the hardest ordeals is when the patient doesn’t return at the agreed time”, one of them says. An indelible impression that participants recall from their professional experience is the encounter with their own anxiety when a patient who had attempted suicide is preparing to go on the first leave: “It is very unpleasant. … to dare to ask …, I was afraid of making a mistake.” The participants describe how they work to ensure good transitions and relieve family members of the responsibility when it is too heavy to bear, as this statement illustrates:

We always speak with them about the first leave from the ward. If the family members seem uncertain and find it difficult to tell the patient, we take responsibility for making the decision that it is too early for the first leave.

Some of the participants refer to prearranged telephone contact with the patient on leave, and the length of a first leave is curtailed if the patient or family members become insecure. Joint conversations with the patient and relatives often discuss the first leaves of absence from the hospital. The participants have experienced that conversations like these sometimes open for more direct communication between patient and family.

Participants mention tentative discharge dates are set early on, and the family members are informed. If the patient himself or the family think that it is too early for discharge, the staff members frequently take “another round” of discussion. Situations in which patients do not want to give the names of their family members because of complicated relationships or to spare the family from worry make cooperation difficult. However, as several participants point out, “This frequently changes after things calm down.”

The professional network is involved in transitions to other care services or discharge to return home. The participants are committed to ensuring that the patient already has “the next appointment in his hand” when he is released, as well as getting in contact with the persons who will be following up the patient. They also try to facilitate meetings between the patient and follow-up personnel before discharging a patient from the care facility.

Several of the participants talked about using the crisis plan as a tool for meeting challenges and crises after discharge. The crisis plan describes what the patient can do himself/herself, what others can do, whom they can contact, and the normal signals that commonly come before a crisis. Theese signals that commonly occur before a crisis are important for family members to be aware of. Occasionally, health personnel get input from family members that can be included in such a plan.

Not everyone wants such a plan, some patients put the plan in a drawer, while others post it on the refrigerator door, one of the participants says:

But just drawing up a crisis plan accomplishes something important, because they reflect a little over what will happen when they get sick. They become a little more aware, maybe, just by drawing up the plan, I think.

When patients already have a plan like this from a previous admission, the staff members look at it along with the patient and then make necessary adjustments. The participants report that a procedure more common than involving family members in drawing up the crisis plan is to provide them with a copy when the patient is discharged.

## Interpretative discussion

The results in this study are about challenges, but most of all about opportunities in the encounter with relatives of patients at risk of suicide. In terms of understanding responsibilities and tasks when encountering family members, health personnel see *acknowledgement as a premise for involving family members* and as a basic and effective attitude. This is in line with Schibbye’s (2009) description of acknowledgement.

Suicidal patients experience a feeling of being acknowledged and valued in contact with health personnel (Berg et al., 2017; Hagen et al., 2018; Vatne & Nåden, 2016). Likewise, acknowledgement can influence the family member’s feeling of being significant, and may perhaps also relieve burdens resulting from responsibility, pressure, helplessness, and guilt (McLaughlin et al., 2014). However, as the health personnel point out, acknowledgement can be perceived as consolement only when it is communicated credibly. McLaughlin et al. (2016) found that family members of suicidal patients lacked validation and acknowledgement in their encounter with health personnel. Communication can be influenced by the attitude of health personnel, for example, by labelling family members as “difficult” or “over-involved” rather than treating them as a resource. The mention in some interviews of “over-involved family members” is usually linked with cases involving young people and particularly mothers, where the dynamics seem to hinder the patient’s improvement. The expression is associated with empathy and a certain projection; interviewees reflect over how they, as parents, might have managed to avoid over-involvement if the patients were their own offspring.

With a perception of the family members as equal partners in collaboration, the health personnel follow up by exploring the family members’ knowledge of the patient, which “far surpasses what we know”. To view the family members as resources entails being receptive to their understanding of the situation and including this in the holistic picture as well as allowing it to have an impact on the planning of subsequent treatment. Studies of immediate family members’ experiences following suicide show that they appreciate being involved and to be there for the patient during recovery (Sellin et al., 2017). This is also recommended as one of the strategies in the Norwegian action plan in Prevention Suicide (Norwegian Ministry of Health and Care Services, 2020). The health personnel in the present study believe that involvement of patients’ family members is a sign that their experiential knowledge is valued. At the same time, the extent to which they are actively included in patients’ treatment process varies. Inclusion appears to be dependent on the treatment, personnel’s attitudes and commitment, the initiative taken by the family members and the patient’s own wishes and needs.

Involvement of family members entails a holistic conception of the patients in their social context, i.e., where they live their individual lives. In this study, involvement of family members might be understood as a responsibility and an act of caregiving by health personnel that responds to the patient’s needs. This requires what Eriksson describes as a presence: “I was there, I saw, I knew, and I became responsible” (Eriksson, K, 2018, p. 15), which seems to be characteristic of the health personnel in this study in terms of their relationship with family members.

Family members of a person at risk of suicide live under emotional pressure, where the sense of responsibility for the life or death of another person is perhaps the weightiest burden for family members. The struggle to maintain the mask towards the outside world (McLaughlin et al., 2014) can aggravate loneliness and worsen suffering (Eriksson, K, 2018). Health personnel claim that one of the most important tasks in the encounter with the suffering of family members, is *embracing their feelings and reactions*. The health personnel are witnesses to family members’ suffering, and this seems to awaken an ethical responsibility in them (Eriksson, K, 2018). Health personnel must take the perspective of both the patients and the family members. To embrace in situations where the involvement of family members is on a collision course with the patient’s desire for distance “is the most difficult thing to deal with”, one of the health personnel says. A study by Weimand et al. (2014) reveals that nurses encountered difficulties associated with the duty of confidentiality and the requirement that the patient must provide consent to inform and involve family members. Legislative regulations, however, set no limits on listening to family members if the latter have been informed that the patient has been admitted. Setting aside a time and place for sharing worries and anxieties can alleviate the family members’ care burden. The health personnel in the present study said that patients normally provided the names of their family members after the acuteness of the situation had abated. In certain situations, on the other hand, health personnel spent much time explaining to the patient why they thought it was important to inform the family members. When conflicts of interest arise, as Pedersen and Weimand (2017) point out, it becomes necessary to reflect critically and discuss what is in the best interest of the patient versus the needs of the family members, along with what should be done in the specific situation.

Embracing family members’ feelings and reactions is linked in the present study not only to the patient’ perceptions, but often to the system and to the decisions that are contrary to what they feel the patient needs. The study by McLaughlin et al. (2016) reports that family members feel they are not sufficiently acknowledged, informed, or supported. The health personnel in the present study seem to spend much time “embracing with the family members”, despite the fact that extra time is not allocated to working with next of kin and that this work cannot be documented to the same extent as patient caregiving. They appear to work in line with what is expected of them in their encounter with family members’ despair, exasperation, exhaustion, fury, and frustration at the prioritization of ethics as a guide (Eriksson, K, 2018) over time and regulatory frameworks.

The theme *Strengthening hope in a situation entailing a serious risk of suicide* is deemed one of the four findings in this study. Family members of a suicidal person are worn down by the fear of the potential death, despair in this situation, loneliness, and the perceived burden of responsibility for the life of a loved one. They are in a state of ongoing suffering that further exacerbates hopelessness. “If there is one thing family members need, it is hope”, one of the health personnel stated. This assertion is in line with Eriksson, K’s (2018) and also reported by Valle and Lohne (2020), that to alleviate suffering, hope is needed. In situations where a patient is still contemplating suicide as a viable option, the family member’s fear derives from a tangible risk of suicide. Hope is uncertain, but at the same time attainable, according to the definition of St. Thomas Aquinas (Herrestad, 2009). Hope is realistic based on an understanding of suicidal ideation as a normal reaction to demanding life events that are perceived as indomitable (Beskow et al., 2013; Brown et al., 2005; Williams, 2014). One common characteristic among suicidal persons is the quest for a way out of unbearable pain and hopelessness (Shneidman, 1998). They seek relief and hope rather than death (Vatne & Nåden, 2014, 2016). The study by Lohne and Severinsson (2004) shows that hope, for patients, is a necessity, not least in crises when uncertainty and fear cast a pall over the attainment of hope. Hope can alleviate despair in both the patient and the family member. The latter need the hope that the patient may regain the will to live.

Family members struggle with helplessness and feelings of guilt over not being able to help a suicidal person who is close to them. This is in line with McLaughlin et al. (2014). Despair and helplessness may lead them into a state of hopelessness. In the present study, health personnel experience that when a patient is admitted, many family members find new hope, namely that treatment will succeed. The health personnel’s own hope draws on their experiences from the psychiatric ward when they convey their confidence to the family members that treatment and care will help, that “things will be better”.

The health personnel’s understanding of the complexity of the concept of hope is important to enable them to convey hope in a manner that alleviates suffering. Hope is primarily relational (Herrestad & Biong, 2010), and health personnel, therefore, must develop their own hope (Moore, 2005). The health personnel’s hope is perceived by the next of kin as meaningful when a loved one is in a critical situation (Valle & Lohne, 2020). Family members of persons at immediate risk of suicide need hope that is future-oriented and can reinforce the belief that the patient and his/her life will improve, and that their efforts to promote improvement are important. One participant noted that the opportunity to construct a more long-term hope in the encounter with a suicidal patient is limited in emergency psychiatric intervention. On the other hand, health personnel described how they observe and convey the specific progress they see in the patient in order to reinforce hope in a situation that is still uncertain. Perhaps being helped to see resources and the “small steps” along the way to recovery can serve as an impulse that can steer the patient towards an attitude of involvement and can inspire hope (Cutcliffe & Barker, 2002). This does not exclude talking with the family members about the risk of suicide and removing their uncertainty and fear. Suffering can be reversed into a positive force only by conveying it to another person, Eriksson says (Eriksson, K, 2018, p. 380). Sharing provides a certain consolation that can strengthen hope.

The theme *Providing reassurance to family members in transitional situations* is understood as caregiving with a relational as well as a practical dimension. The time period encompassing discharge from hospital is considered a risk phase. In a study on suicide conducted in the Agder counties of Norway in the period of 2004–2013 in the field of mental health protection and interdisciplinary specialized treatment for substance abuse, it was found that 14 of 339 suicides occurred within seven days after discharge and that 30 suicides occurred within 30 days (Haaland et al., 2017). Measures are described for ensuring that the situation arising from leaves of absence and discharges is as safe as possible for both the patient and his/her family members. These measures seem to be in line with the patient safety programme (Norwegian Ministry of Health and Care Services, 2014) under the prioritized initiative “suicide prevention in 24-hour emergency psychiatric wards”.

The results include accounts of suicide that occurred during temporary leave, despite all preventive measures including suicide risk assessment were followed. The family members of patients at risk of suicide must be involved in decision-making processes to a far greater extent than is done now, in order to decide whether the time is right and it is sufficiently safe to grant leaves of absence (Leavey, et al., 2017). Based on the results, the family members are informed, but to a lesser degree involved in the decision-making. Taking the pulse of health personnel’s own fear when responsibility is given back to the patient, as described in the results, perhaps makes them better at identifying and understanding the family members’ fear. There is implicit trust in expressing oneself. But trust that is accepted with any reciprocal attitude other than trust develops into mistrust (Martinsen, 2005). The family members’ trust can be reciprocated in ways that make them feel secure, such as postponing a temporary leave that the patient has been looking forward to and thereby sparing the family from the patient’s disappointment or anger by being the ones who made the decision. Health personnel can also enter into dialogue with both parties and negotiate agreements that reassure the family members in this situation.

One way to reassure the patient and the family members in a transitional situation is to draw up a crisis plan for coping, the health personnel noted. According to the Norwegian Ministry of Health and Care Services (2014), a crisis plan must be drawn up prior to a safe discharge of the patient. Difficulties arise when patients do not want such a plan or when they refuse to identify their close family members. The patient’s shame (reference to be inserted; McLaughlin et al., 2014) makes it difficult to involve family members. Participation of family members in drawing up an individualized crisis plan, can help to reduce their uncertainty or feeling of helplessness, and it can help to clarify responsibility between the parties. The feeling of responsibility may burden the family members even when the patient is experiencing a period of improvement and is assessed as having suicidal impulses under control. Psychoeducation has proven to help family members better understand suicidality (Sun et al., 2013) by helping them to understand how they themselves are affected by another’s suicidality and by making it easier for them to cope in this kind of relationship. Precisely the initiative and effort of close family and friends were found to be decisive in the struggle to regain the will to live after an attempted suicide. Results from another study showed, however, that family members were rarely involved (Vatne & Nåden, 2014).

The results from the present study confirm the need to involve family members as well as the patients’ professional care network in the treatment process, including in situations where the risk of suicide is connected with conflicts with the closest family relatives. Such collaboration is in line with Mellesdal et al. (2010) to help patients gain constructive coping strategies and break patterns of suicidal behaviour and readmission and to potentially reinforce the family members’ sense of security.

### Methodological considerations

Conversation is a tool to create valid knowledge in qualitive interviews Fog (2004); Kvale and Brinkmann (2009). Data in this study were produced in an atmosphere characterized by openness and trust. But as Fog (2004) writes, the researcher must always listen to the “noise” that can occur in the communication between themselves and the participant. Data, based on content and depth, were considered rich enough to answer the research question after interviews with 12 participants, recruited from staff members in two hospitals. Recruiting participants from several hospitals could possibly have added more nuances to the themes.

Findings in this study are considered valid and reliable, based on the chosen methodology and theoretical perspectives. In line with Kvale and Brinkmann (2009), to validate is to reflect and control through all stages in the research process.

### Implications for practice

Based on the perspectives of the health personnel who participated in our study, we are of the opinion that the findings can offer inspiration and ideas for good encounters with suicidal patients’ next of kin. Research has documented how health personnel think and relate to this specific group of family members: embracing reactions, strengthening hope, and seeking to provide reassurance in transitional situations. Acknowledgement is the basic attitude at the core of good encounters with family members. In this study, health personnel considered relatives as resources, which lays the foundation for a collaboration on the suicidal patient’s treatment and recovery process.

## Summarized understanding and conclusion

In this study, caregiving for family members of patients at risk of suicide is linked with a holistic view having various interacting dimensions (Eriksson, K, 2018), and where the patient and his/her closest kin influence one another mutually, positively, or negatively. Conceptualizing family and networks as resources, as seems to be the strategy of the participants in the study, harbours a belief in a latent potential. The experiences and perceptions of the family members may portray the patient’s situation holistically, provided they are met with openness. To be open and receptive to another’s understanding, according to Gadamer (2004), requires that we relate to the other’s perception, while at the same time remaining receptive to the other’s conception of the whole.

The family members’ suffering derives from the despair, fear, and insecurity caused by the patient’s seeing suicide as a solution in a difficult life situation. Suffering is acknowledged when it is shared (Eriksson, K, 2018). When one embraces and meets suffering with consolation, as the informants in this study describe, the family members’ hope can be strengthened. Being a witness to the suffering and needs of the family members is understood, in the light of Eriksson, K (2018), as awakening responsibility in the participants in this study, who then initiate different acts of caregiving. In terms of content, in line with Martinsen (2005), the components of caregiving seem to have a moral, relational and practical dimension. Trust is essential in caring for family members and the patient, and it is a premise for involvement of and increased cooperation with the family members, in the best interests of the patient.

Caregiving’s main components as they are conceptualized in this study include listening, embracing, strengthening hope, reassuring family members—all in the light of acknowledgement of the other as a basic attitude. The study visualizes ways by which health personnel take ethical and professional responsibility in the encounter with family members of patients at risk of suicide, despite framework conditions and resources that do not appear to be commensurate with the responsibility and effort of the tasks they undertake.
